# Supplementary material for: Plants with higher dispersal capabilities follow ‘abundant-centre’ distributions but such patterns remain rare in animals
Source: Nat Commun. 2025 Sep 2;16:8205. doi: 10.1038/s41467-025-63566-0 (PMC12405579; doi:10.1038/s41467-025-63566-0)
Supplement: Supplementary file 1 — Supplementary Information [file 41467_2025_63566_MOESM1_ESM.pdf]

Supplementary information for: Panter *et al*. 2025. Plants with higher dispersal capabilities follow 'abundant-centre' distributions but such patterns remain rare in animals**.** *Nature Communications***.**

SUPPLEMENTARY TABLES

Table S1. An overview of species trait justification, supporting reference and a priori expectations on global abundance–distance relationships in animals and plants.

Traits/moderators	Taxa	Justification of inclusion	Reference	*A priori* expectation
Morphological				
Group (categorical)	Animals	Dispersal capability differs between animal groups.	Bohonak[1]	Groups that are better able to track changing environments, e.g., birds, will conform more to abundant-centre patterns.
$\log_{10}$ Body size	Animals	Body size increases linearly with dispersal capability.	Stevens et al.[2]	Larger-bodied animals will conform more to abundant-centre patterns as they are better able to track changing environments.
$\log_{10}$ Mean plant height (m)	Plants	Dispersal capability is correlated with plant height.	Thomson et al.[3]	Relative to smaller plants, taller plants are better dispersers and will conform more to abundant-centre patterns.
Ecological				
Functional group (categorical)	Plants	Dispersal capacity may be intrinsically linked with functional group, depending on the environment.	Aslan et al.[4]	Faster growing and shorter-lived species, e.g., grasses and herbs, will conform more to abundant-centre patterns, as they are better able to track changing environments.
Invasive status (1,0)	Both	Invasion fronts may benefit invasive species when dispersing.	Arim et al.[5]	Invasive species will not conform to abundant-centre patterns, as abundant-edge distributions indicate invasion fronts.
Life span (categorical)	Plants	Shorter-lived species may be able to track changing environments more easily.	Beckman et al.[6]	Shorter-lived plants, e.g., annuals and biennials, will conform more to abundant-centre patterns.
Life form (categorical)	Plants	Following Raunkiær's system, the location of the growing bud may reflect a species' dispersal ability as these relate directly to the environment.	Beckman et al.[6]	Geophytes, therophytes, and hemicryptophytes will conform more to abundant-centre patterns, as they tend to be faster-growing and shorter-lived.

$\log_{10}$ Seed mass (mg)	Plants	Small-seeded species should disperse better than large-seeded species.	Venable & Brown[7]	Plants with smaller seeds will conform more to abundant-centre patterns, as they are better able to track changing environments.
Feeding guild (categorical)	Animals	Feeding guild, particularly omnivores, influences dispersal capability.	Stevens et al.[2]	Omnivorous species will conform more to abundant-centre patterns, as they are better able to track changing environments. Expected interaction effect between body size and feeding guild.
Geographical				
$\log_{10}$ Range size (km^2)	Both	Dispersal capability can be an important process moderating range size.	Lester et al.[8]	Species with larger ranges are better able to track changing environments will conform more to abundant-centre patterns.
Absolute latitude (°)	Both	Terrestrial species distributed at higher latitudes may be better at tracking changing environments due to increased seasonality away from the equtor.	Ruggiero & Werenkraut[9]	Abundant-centre patterns should be more prominent in species that are distributed at higher latitudes.

Table S2. Morphological, ecological and geographical species traits (hereafter 'moderators') used to explore the effects on 3,660 abundance–distance relationships. Traits presented with their subgroups and units, inclusion within each taxonomic group, and trait data sources. Note, data for 3,060 animal and 600 plant species were used in the statistical analyses.

Traits/moderators	Subgroups/units	Taxonomic group		References
		Animals	Plants	
Morphological				
Group	birds, freshwater fishes, mammals and reef fishes	✓		N/A
Body size	centimetres; grams (cm; g)	✓		Froese & Pauly[10]; Tobias et al.[11]; Cooke et al.[12]
Mean plant height	metres (m)		✓	Kattge et al.[13]
Ecological				
Functional group	grasses, herbs, shrubs and trees		✓	Kühn et al.[14]
Invasiveness	binary (1,0)	✓	✓	https://www.iucngisd.org/gisd/
Life span	annuals, annuals/biennials, biennials, biennials/perennials, perennials		✓	Kühn et al.[14]
Life form	geophytes, therophytes, hemicryptophytes, phanerophytes		✓	Kühn et al.[14]
Seed mass	milligrams (mg)		✓	Kattge et al.[13]
Feeding guild	carnivores, omnivore, herbivores	✓		Froese & Pauly[10]; Cooke et al.[12]
Geographical				
Range size	square kilometres (km^2)	✓	✓	Froese & Pauly[10]; Tobias et al.[11]; Sporbert et al.[15]; Santini et al.[16]; Shalom et al.[17]
Absolute latitude	degrees (°)	✓	✓	Enquist et al.[18]; Froese & Pauly[10]; Tobias et al.[11]; Cooke et al.[12]; Sporbert et al.[15]; Santini et al.[16]

Table S3. Model results for the intercept-only grand mean effects for 3,060 animal and 600 plant abundance–distance relationships (Fishers' *z*-scores). Grand mean models represent linear effects models, weight weighted by the logarithm of the number of abundance observations per species. SE = standard error, CI = confidence interval. Source data are provided as a Source Data file.

Data set	Estimate	SE	*t*	*p*	95% CI (lower)	95% CI (upper)
Animals (N = 3,060 spp.)						
(Intercept)	0.006	0.004	1.447	0.148	-0.002	0.014
Plants (N = 600 spp.)						
(Intercept)	-0.079	0.005	-15.643	0.000	-0.089	-0.069

Table S4. Outputs from the weight linear effects models with proportional variance explained from the most parsimonious animal and plant models, exploring the effects of species traits and geographic variables on abundance–distance relationships for 3,060 animal and 600 plant species. Significant predictors in **bold**. SE = standard error, CI = confidence interval. Source data are provided as a Source Data file.

Fixed effects	Estimate	SE	*t*	*p*	R^2
Animals (N = 3,060)					0.03
**(Intercept)**	**-0.040**	**0.006**	**-6.533**	**7.51E-11**	
Group(freshwater fishes)	0.008	0.038	0.222	0.8241	
**Group(mammals)**	**0.076**	**0.021**	**3.688**	**0.0002**	
**Group(reef fishes)**	**0.081**	**0.008**	**9.580**	**1.93E-21**	
**$\log_{10}$ Range size (km^2)**	**0.009**	**0.004**	**2.232**	**0.0257**	
Plants (N = 600)					0.14
**(Intercept)**	**-0.157**	**0.027**	**-5.911**	**5.73E-09**	
functional group(herb)	0.010	0.015	0.661	0.5088	
**functional group(shrub)**	**0.085**	**0.025**	**3.386**	**0.0008**	
**functional group(trees)**	**0.074**	**0.025**	**2.925**	**0.0036**	
**Invasive status(non-invasive)**	**0.060**	**0.023**	**2.585**	**0.0100**	
**Absolute latitude (°)**	**-0.035**	**0.008**	**-4.428**	**1.13E-05**	
**$\log_{10}$ Seed mass (mg)**	**-0.015**	**0.007**	**-2.117**	**0.0346**	

Table S5. Outputs from the weight linear effects models with proportional variance explained from the most parsimonious animal and plant interaction models, exploring the effects of species trait and geographic variable combinations on abundance–distance relationships for 3,060 animal and 600 plant species. Significant predictors in **bold**. SE = standard error, CI = confidence interval. Source data are provided as a Source Data file.

Fixed effects	Estimate	SE	*t*	*p*	95% CI (lower)	95% CI (upper)	R^2
Animals (N = 3,060)							0.05
(Intercept)	-0.021	0.027	-0.777	0.437	-0.073	0.032	
**Feeding guild(herbivores)**	**-0.052**	**0.018**	**-2.862**	**0.004**	**-0.088**	**-0.016**	
Feeding guild(omnivores)	-0.005	0.013	-0.380	0.704	-0.031	0.021	
Group(freshwater fishes)	-0.132	0.299	-0.442	0.658	-0.719	0.454	
**Group(mammals)**	**0.368**	**0.100**	**3.671**	**0.000**	**0.171**	**0.564**	
**Group(reef fishes)**	**0.147**	**0.034**	**4.338**	**0.000**	**0.081**	**0.213**	
$\log_{10}$ Body size (cm; g)	-0.011	0.013	-0.825	0.409	-0.037	0.015	
Absolute latitude (°)	0.000	0.001	-0.509	0.610	-0.002	0.001	
Feeding guild(herbivores) × Group(freshwater fishes)	-0.439	0.284	-1.543	0.123	-0.997	0.119	
Feeding guild(omnivores) × Group(reshwater fishes)	0.133	0.093	1.427	0.154	-0.050	0.316	
Feeding guild(herbivores) × Group(mammals)	0.097	0.063	1.541	0.123	-0.026	0.220	
Feeding guild(omnivores) × Group(mammals)	-0.092	0.064	-1.427	0.154	-0.217	0.034	
Feeding guild(herbivores) × Group(reef fishes)	0.044	0.023	1.881	0.060	-0.002	0.090	
Feeding guild(omnivores) × Group(reef fishes)	-0.021	0.020	-1.048	0.295	-0.060	0.018	
Group(freshwater fishes) × $\log_{10}$ Body size (cm; g)	0.179	0.112	1.598	0.110	-0.041	0.399	
**Group(mammals) × $\log_{10}$ Body size (cm; g)**	**-0.054**	**0.018**	**-3.098**	**0.002**	**-0.089**	**-0.020**	
Group(reef fishes) × $\log_{10}$ Body size (cm; g)	-0.003	0.019	-0.156	0.876	-0.040	0.034	
Group(freshwater fishes) × Absolute latitude (°)	-0.004	0.006	-0.747	0.455	-0.016	0.007	
**Group(mammals) × Absolute latitude (°)**	**-0.005**	**0.002**	**-2.862**	**0.004**	**-0.008**	**-0.002**	
**Group(reef fishes) × Absolute latitude (°)**	**-0.003**	**0.001**	**-5.258**	**0.000**	**-0.005**	**-0.002**	
$\log_{10}$ Body size (cm; g) × Absolute latitude (°)	0.000	0.000	1.135	0.257	0.000	0.001	
Plants (N = 600)							0.16
(Intercept)	0.107	0.066	1.614	0.107	-0.023	0.236	
Functional group(herbs)	0.009	0.015	0.603	0.547	-0.020	0.039	
**Functional group(shrubs)**	**0.104**	**0.032**	**3.217**	**0.001**	**0.041**	**0.167**	
Functional group(trees)	0.049	0.035	1.394	0.164	-0.020	0.117	
**Invasive status(non-invasive)**	**0.063**	**0.023**	**2.714**	**0.007**	**0.017**	**0.108**	
$\log_{10}$ Seed mass (mg)	0.016	0.041	0.396	0.692	-0.064	0.096	
**Absolute latitude (°)**	**-0.005**	**0.001**	**-4.821**	**0.000**	**-0.007**	**-0.003**	
Life form(hemicryptophytes)	-0.025	0.027	-0.920	0.358	-0.077	0.028	
Life form(phanerophytes)	-0.020	0.032	-0.637	0.524	-0.083	0.042	
Life form(therophytes)	-0.025	0.027	-0.900	0.368	-0.078	0.029	
Functional group(herbs) × $\log_{10}$ Seed mass (mg)	0.013	0.035	0.383	0.702	-0.055	0.082	

Functional group(shrubs) × log_{10} Seed mass (mg)	-0.089	0.041	-2.191	0.029	-0.168	-0.009
Functional group(trees) × log_{10} Seed mass (mg)	0.013	0.035	0.370	0.711	-0.056	0.082
Invasive status(non-invasive) × log_{10} Seed mass (mg)	-0.036	0.023	-1.559	0.120	-0.081	0.009

Table S6. Underlying abundance data sources used by the 14 studies included in this synthesis, by taxonomic group and ordered by number of species. Abundance data sources presented separately for animals (3,329 species) and plants (699 species). Note, data for 3,060 animal and 600 plant species were used in the statistical analyses.

Study	Taxa	N spp.	Abundance data source	Reference
**Animals**				
Dallas et al.[19]	Birds	1677	eBird	Sullivan et al.[30]
	Mammals	125	Mammal Community Database (MCDB)	Thibault et al.[31]
	Freshwater fishes	46	The United States Environmental Protection Agency Environmental Monitoring and Assessment Program (EPA-EMAP) and the National Water Quality Assessment (NAWQA)	https://www.epa.gov/emap/; Knouft & Anthony[32]
Shalom et al.[17]	Reef fishes	1235	Reef Life Survey and the General Approach to Species Abundance Relationships Database (GASPAR)	https://reeflifesurvey.com/; Kulbicki et al.[33]
Freeman & Beehler[20]	Birds	129	Collected by authors	Freeman & Beehler[31]
Santini et al.[16]	Birds	8	TetraDENSITY Database	Santini et al.[16]
	Mammals	96		
Feldman et al.[21]	Birds	6	North American Waterfowl Breeding Population and Habitat Survey (BPOP) and the North American Breeding Bird Survey (BBS)	Zimpfer et al.[34]; Sauer et al.[35]
Wen et al.[22]	Mammals	5	Collected by authors	Wen et al.[22]
Martínez-Gutiérrez et al.[23]	Mammals	1	Literature search	Martínez-Gutiérrez et al.[23]
Chaiyes et al.[24]	Mammals	1	Global Biodiversity Information Facility, VertNet Database and Mammal Species of the World	GBIF.org; http://www.vertnet.org; Smithsonian National Museum of Natural History
**Plants**				
Sporbert et al.[15]	Herbs, dwarf shubs and shrubs	532	European Vegetation Archive (EVA)	Chytrý et al.[36]
Dallas et al.[19]	Trees	162	USDA Forest Inventory Analysis Database (USDA FIA)	Woudenberget et al.[37]
Phiri et al.[25]	Dwarf shrub	1	Collected by authors	Phiri et al.[25]
McMinn et al.[26]	Herb	1	Collected by authors	McMinn et al.[26]
Dixon et al.[27]	Herb	1	Collected by authors	Dixon et al.[27]
Baer & Maron[28]	Herb	1	Collected by authors	Baer & Maron[28]
Gao et al.[29]	Tree	1	Collected by authors	Gao et al.[29]

SUPPLEMENTARY METHODS

Effects of underlying abundance data sources on abundance–distance relationships

To explore the effects of underlying abundance data source on global abundance–distance relationships, we compiled a new variable "underlying data source". We ran an additional linear model, weighted by the logarithm of the number of observations per species, with the Spearman rank correlation coefficients fitted as the response term and the "underlying data source" variable fitted as an explanatory fixed effect term. Abundance–distance rank correlation coefficients were transformed to Fisher's *Z* scores (to achieve approximate normality, hereafter 'effect sizes'). We quantified the underlying data source subgroup-level average effect sizes with estimated marginal mean effects using the *ggeffects* package[38].

Table S7. Outputs from the weight linear effects models exploring the effects of underlying data source on global abundance-distance relationships (Fishers' *Z*) for 3,060 animal and 600 plant species. Significant effects highlighted in bold. SE = standard error and CI = confidence interval. Source data are provided as a Source Data file.

Data Source	Estimate	SE	*t*	*p*	95% CI (lower)	95% CI (upper)	R^2
Animals (N = 3,060)							0.03
(Intercept)	-0.104	0.130	-0.802	0.423	-0.359	0.151	
Collected by authors	0.139	0.133	1.048	0.295	-0.121	0.400	
eBird	0.062	0.130	0.474	0.635	-0.194	0.317	
EPA-EMAP; NAWQA	0.078	0.135	0.574	0.566	-0.188	0.343	
GBIF; VertNet; Mammal Species of the World	-0.030	0.309	-0.098	0.922	-0.636	0.576	
Literature Search	-0.273	0.282	-0.967	0.334	-0.825	0.280	
MCDB	0.154	0.133	1.156	0.248	-0.107	0.414	
Reef Life Survey; GASPAR	0.149	0.130	1.145	0.252	-0.106	0.404	
TetraDENSITY	0.092	0.134	0.687	0.492	-0.170	0.353	
Plants (N = 600)							0.10
**(Intercept)**	**-0.096**	**0.005**	**-18.363**	**0.000**	**-0.107**	**-0.086**	
**USDA Forest Inventory and Analysis database**	**0.104**	**0.013**	**8.143**	**0.000**	**0.079**	**0.130**	

Data source abbreviations: EPA-EMAP = The United States Environmental Protection Agency- Environmental Monitoring and Assessment Program; NAWQA = The National Water-Quality Assessment; MCDB = Mammal Community Database; GASPAR = General Approach to Species Abundance Relationships Database; USDA FIA = USDA Forest Inventory and Analysis Database.

The underlying data source explained approximately 3% and 10% of variation within the animal and plant data sets, respectively. There were significant effects of the underlying data source in both animal ($F_{1,3051}$ = 14.364, p < 0.0001) and plant ($F_{1,598}$ = 66.302, p < 0.0001) data sets. There was a significant positive effect for the USDA Forest Inventory Analysis (from Dallas et al.[19]), suggesting no support for abundant-centre patterns in North American trees.

Effects of migration strategies on abundance–distance relationships

Using a subset data set for 1,638 bird species, we explored the effects of migration strategies on abundance–distance relationships. Migration strategy data was obtained from the AVONET Database[11], and comprised of the following categorical levels: 1) 'sedentary' - species does not migrate, 2) 'partially' – partially migratory, a minority of the population migrates long distances, or most of the population undergoes short-distance migration, nomadic movements, distinct altitudinal migration, and 3) 'migratory' – whereby the majority of the population undertakes long-distance migration[39]. An additional linear model was run, weighted by the logarithm of the number of observations per species, with the abundance–distance rank correlation coefficient fitted as the response term and the migration strategy variable fitted as an explanatory fixed effect term.

On average, avian abundance–distance relationships were negative ($\bar{x}$ = -0.037 ± 0.255 [±SD]) indicating an abundant-centre pattern. However, migration strategy had a non-significant effect on bird abundance–distance relationships ($F_{1,1680}$ = 0.526, p = 0.591, R^2 = < 0.001; Table S9).

Table S8. Outputs from the weight linear effects model exploring the effects of migration strategy on global abundance–distance relationships (Fishers' *Z*) for 1,683 bird species. Significant effects highlighted in **bold**. SE = standard error and CI = confidence interval. Source data are provided as a Source Data file.

Fixed effect	Estimate	SE	*t*	*p*	95% CI (lower)	95% CI (upper)	R^2
							< 0.001
**(Intercept)**	**-0.031**	**0.011**	**-2.840**	**0.005**	**-0.053**	**-0.010**	
Migration(partially)	-0.010	0.019	-0.517	0.605	-0.047	0.027	
Migration(sedentary)	-0.014	0.014	-1.024	0.306	-0.041	0.013	

Modelling the effects of wing morphology on abundance–distance relationships in birds

Wing morphology has been found to be an importer predictor of dispersal distance in birds[40,41]. Therefore, similarly to the bird migration analysis, we explored the effects of the Hand-Wing Index (HWI) from the AVONET database for 1,683 bird species. We ran an additional linear model, weight by the logarithm of the number of observations per species, with the avian abundance–distance correlation coefficients fitted as the response term and the HWI variable fitted as a predictor variable. There was no significant effect of wing morphology on abundance–distance relationships in birds ($F_{1,1681}$ = 0.0004*,* p = 0.986, R^2 = < 0.001; Table S10), suggesting that avian dispersal capability does not affect abundance–distance relationships in geographic space.

Table S9. Outputs from the weight linear effects model exploring the effects of wing morphology (using Hand-Wing Index as a proxy) on global abundance–distance relationships (Fishers' *Z*) for 1,683 bird species. Significant effects highlighted in bold. SE = standard error and CI = confidence interval. Source data are provided as a Source Data file.

Fixed effect	Estimate	SE	*t*	*p*	95% CI (lower)	95% CI (upper)	R^2
							< 0.001
(Intercept)	-0.040	0.013	-3.065	0.002	-0.066	-0.015	
Hand Wing Index	< 0.0001	< 0.0001	0.017	0.986	-0.001	0.001	

Exploring the effects of historical range contractions on global abundance–distance relationships in Late Quaternary mammals

To account for how global abundance–distance relationships manifest in species that have undergone historical range contractions, which have been shown to result in ecological marginalisation[42], we performed additional analyses on a subset of 202 mammalian species from the Late Quaternary. Using historical range data from the PHYLACINE database (version 1.2)[43], we calculated the log ratio between the number of cells within a species' current range and divided this by the number of cells in the species' present natural range. Similar to our other supplementary analyses, we ran an additional linear model, weighted by the logarithm of the number of observations per species, with the abundance–distance rank

correlation coefficient fitted as the response term and the resulting log ratio range contraction variable fitted as an explanatory fixed effect term. There was a positive effect of historical range contraction on mammalian abundance–distance relationships, however, this effect was non-significant ($F_{1,200}$ = 1.242, p = 0.266, R^2 = 0.001; Table S11).

Table S10. Outputs from the weight linear effects model exploring the effects of historical range contractions on global abundance–distance relationships (Fishers' *Z*) for 202 Late Quaternary mammal species. Historical range contractions measured by taking the log ratio of the number of cells in species' current range divided by the number of cells in the species' present natural range[43]. SE = standard error and CI = confidence interval. Source data are provided as a Source Data file.

Fixed effect	Estimate	SE	*t*	*p*	95% CI (lower)	95% CI (upper)	R^2
							0.001
(Intercept)	0.043	0.035	1.247	0.214	-0.025	0.112	
Range contraction	< 0.0001	< 0.0001	-1.115	0.266	-0.0001	0.00003	

Table S11. Initial literature search string used to collate relevant literature on the ISI's Web of Science database (23rd July 2021), with an optimised search string using the *litsearchr* R package[44]. The initial search returned 818 results and the optimized search returned 1,019 results.

Initial search string	Optimized search string
(abundan* OR abundance-cent* OR abundant niche-cent* OR niche cent* OR abundant-centre hypothesis)	((("abund* centr*" OR "centr* hypothesi*" OR "local* abund*" OR "rang* centr*" OR "rang* edges" OR "rang* margin*" OR "speci* abund*")
**AND**	**AND**
(range OR geographic range OR range size OR range edge OR species distribution)	("distribut* rang*" OR "elev* gradient*" OR "geograph* rang*" OR "rang* edge" OR "rang* limit*" OR "rang* size*" OR "speci* distribut*" OR "speci* rang*" OR "abund* centr*" OR "centr* hypothesi*" OR "elev* rang*" OR "geograph* distribut*" OR "local* abund*" OR "rang* centr*" OR "rang* margin*" OR "spatial* distribut*")))

SUPPLEMENTARY FIGURES

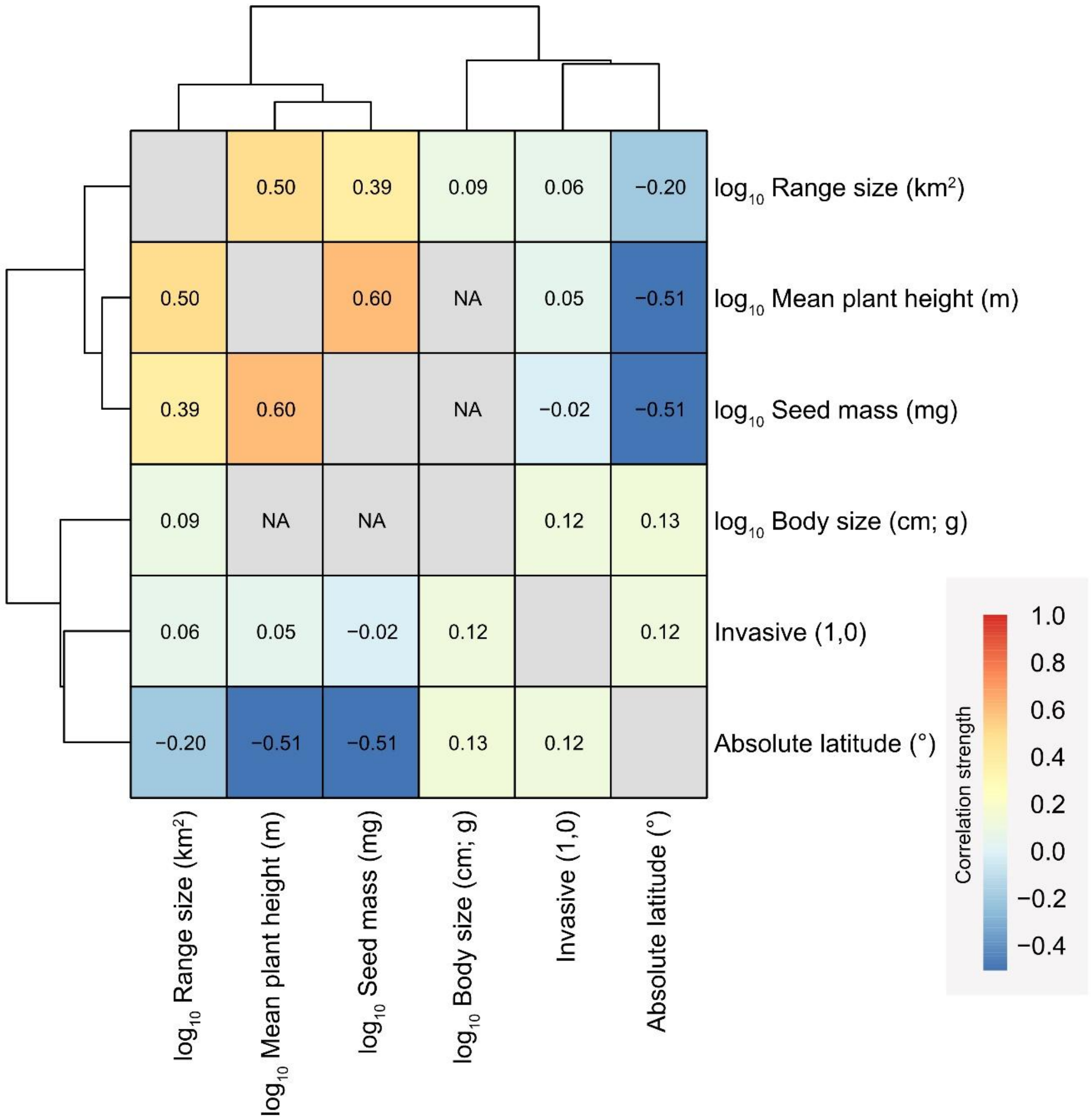

Figure S1. Output from the test of collinearity (Spearman Rank Correlation) between continuous species trait variables fitted as moderators in the series of meta-regression models. Continuous moderators were considered 'colinear' if the Spearman Rank Correlation coefficient ≥ 0.70.

SUPPLEMENTARY REFERENCES

1. Bohonak, A. J. Dispersal, gene flow, and population structure. *Q. Rev. Biol.* **74**, 1 (1999).
2. Stevens, V. M., Whitmee, S., Le Galliard, J- F., Clobert, J., Böhning-Gaese, K., Bonte, D., Brändle, M., Dehling, D. M., Hof, C., Trochet, A. & Baguette, M. A comparative analysis of dispersal syndromes in terrestrial and semi-terrestrial animals. *Ecol. Lett.* **17**, 1039–1052 (2014).
3. Thomson, F. J., Moles, A. T., Auld, T. D. & Kingsford, R. T. Seed dispersal distance is more strongly correlated with plant height than with seed mass. *J. Ecol.* **99**, 1299–1307 (2011).
4. Aslan, C., Beckman, N. G., Rogers, H. S., Bronstein, J., Zurell, D., Hartig, F., Shea, K., Pejchar, L., Neubert, M., Poulsen, J., HilleRisLambers, J., Miriti, M., Loiselle, B., Effiom, E., Zambrano, J., Schupp, G., Pufal, G., Johnson, J., Bullock, J. M., Brodie, J., Bruna, E., Cantrell, R. S., Decker, R., Fricke, E., Gurski, K., Hastings, A., Kogan, O., Razafindratsima, O., Sandor, M., Schreiber, S., Snell, R., Strickland, C. & Zhou, Y. Employing plant functional groups to advance seed dispersal ecology and conservation. *AoBP* **11**, plz006 (2019).
5. Arim, M., Abades, S. R., Neill, P. E., Lima, M. & Marquet, P. A. Spread dynamics of invasive species. *PNAS* **103**, 374–378 (2005).
6. Beckman, N. G., Bullock, J. M. & Salguero-Gómez, R. High dispersal ability is related to fast life-history strategies. *J. Ecol.* **106**, 1349–1362 (2018).
7. Venable, D. L. & Brown, J. S. The selective interactions of dispersal, dormancy and seed size as adaptations for reducing risk in variable environments. *Am. Nat.* **131**, 360–384 (1988).
8. Lester, S. E., Ruttenberg, B. I., Gaines, S. D. & Kinlan, B. P. The relationship between dispersal ability and geographic range size. *Ecol. Lett.* **10**, 745–758 (2007).
9. Ruggiero, A. & Werenkraut, V. One-dimensional analyses of Rapoport's rule reviewed through meta-analysis. *Glob. Ecol. Biogeogr.* **16**, 401–414 (2007).
10. Froese, R. & Pauly, D. FishBase. World Wide Web Electronic Publication. 02/2022 https://www.fishbase.org (2022).
11. Tobias, J. A., Sheard, C., Pigot, A. L., Devenish, A. J. M., Yang, J., Sayol, F., Neate-Clegg, M. H. C., Alioravainen, N., Weeks, T. L., Barber, R. A., et al.

AVONET: morphological, ecological and geographical data for all birds. *Ecol. Lett.* **25**, 581–597 (2022).
12. Cooke, R., Gearty, W., Chapman, A. S. A., Dunic, J., Edgar, G. J., Lefcheck, J. S., Rilov, G., McClain, C. R., Stuart-Smith, R. D., Lyons, S. K. & Bates, A. E. Anthropogenic disruptions to longstanding patterns of trophic-size structure in vertebrates. *Nature Ecol. Evol*. 6, 684–692.
13. Kattge, J., Bönisch, G., Díaz, S. et al. TRY plant trait database – enhanced coverage and open access. *Glob. Change Biol.* **26**, 119–188 (2020).
14. Kühn, I., Durka, W. & Klotz, S. Biolflor – a new plant-trait database as a tool for plant invasion ecology. *Divers. Distrib.* **10**, 363–365 (2004).
15. Sporbert, M., Keil, P., Seidler, G., Bruelheide, H., Jandt, U., Aćić, S., Biurrun, I., Campos, J. A., Čarni, A., Chytrý, M. et al. Testing macroecological abundance patterns: the relationship between local abundance and range size, range position and climatic suitability among European vascular plants. *J. Biogeogr.* **47**, 2210–2222 (2020).
16. Santini, L., Isaac, N.J.B. & Ficetola, G.F. TetraDENSITY: a database of population density estimates in terrestrial vertebrates. *Glob. Ecol. Biogeogr*. **27**, 787–791 (2018b).
17. Shalom, H. Y., Granot, I., Blowes, S. A., Friedlander, A., Mellin, C., Ferreira, C. E. L., Arias-González, J. E., Kulbicki, M., Floeter, S. R., Chabanet, P., Parravicini, V. & Belmaker, J. A closer examination of the 'abundant centre' hypothesis for reef fishes. *J. Biogeogr.* **47**, 2194–2209 (2020).
18. Enquist B. J., Condit R., Peet R. K., Schildhauer M. & Thiers B. M. Cyberinfrastructure for an integrated botanical information network to investigate the ecological impacts of global climate change on plant biodiversity. Preprint at https://doi.org/10.7287/peerj.preprints.2615v2 (2016).
19. Dallas, T., Decker, R. R. & Hastings, A. Species are not most abundant in the centre of their geographic range or climatic niche. *Ecol. Lett.* **20**, 1526–1533 (2017).
20. Freeman, B. G. & Beehler, B. M. Limited support for the "abundant centre" hypothesis in birds along a tropical elevational gradient: implications for the fate of lowland tropical species in a warmer future. *J. Biogeogr.* **45**, 1884–1895 (2018).

21. Feldman, R. E., Anderson, M. G., Howerter, D. W. & Murray, D. L. Where does environmental stochasticity most influence population dynamics? An assessment along a regional core-periphery gradient for prairie breeding ducks. *Glob. Ecol. Biogeogr.* **24**, 896–904 (2015).
22. Wen, Z., Ge, D., Feijó, A., Du, Y., Cheng, J., Sun, J., Wang, Y. & Xia, L. Varying support for abundance-centre and congeneric-competition hypotheses along elevation transects of mammals. *J. Biogeogr.* **48**, 616–627 (2020).
23. Martínez-Gutiérrez, P. G., Martínez-Meyer, E., Palomares, F. & Fernández, N. Niche centrality and human influence predict rangewide variation in population abundance of a widespread mammal: the collared peccary (*Pecari tajacu*). *Divers. Distrib.* **24**, 103–115 (2017).
24. Chaiyes, A., Escobar, L. E., Willcox, E. V., Duengkae, P., Suksavate, W., Watcharaanantapong, P., Pongpattananurak, N., Wacharapluesadee, S. & Hemachudha, T. An assessment of the niche centroid hypothesis: *Pteropus lylei* (Chiroptera). *Ecosphere* **11,** e03134 (2020).
25. Phiri, E. E., McGeoch, M. A. & Chown, S. L. The abundance structure of *Azorella selago* Hook. f. on sub-Antarctic Marion Island: testing the peak and tail hypothesis. *Polar Biol.* **38**, 1881–1890 (2015).
26. McMinn, R. L., Russell, F. L. & Beck, J. B. Demographic structure and genetic variability throughout the distribution of platte thistle (*Cirsium canescens* Asteraceae). *J. Biogeogr.* **44**, 375–385 (2016).
27. Dixon, A., Herlihy, C. R. & Busch, J. W. Demographic and population-genetic test provide mixed support for the abundant centre hypothesis in the endemic plant *Leavenworthia stylosa*. *Mol. Ecol.* **22**, 1777–1791 (2013).
28. Baer, K. C. & Maron, J. L. Declining demographic performance and dispersal limitation influence the geographic distribution of the perennials forb *Astragalus utahensis* (Fabaceae). *J. Ecol.* **107**, 1250–1262 (2019).
29. Gao, W- Q., Ni, Y- Y., Xue, Z- M., Wang, X- F., Kang, F- F., Hu, J., Gao, Z- H., Jiang, Z- P. & Liu, J- F. Population structure and regeneration dynamics of *Quercus variabilis* along latitudinal and longitudinal gradients. *Ecosphere* **8**, e01737 (2017).
30. Sullivan, B. L., Wood, C. L., Iliff, M. J., Bonney, R. E., Fink, D. & Kelling, S. eBird: a citizen-based bird observation network in the biological sciences. *Biol. Conserv*. 10, 2282–2292 (2009).

31. Thibault, K. M., Supp, S. R., Giffin, M., White, E. P. & Ernest, S. K. M. Species composition and abundance of mammalian communities. *Ecol*. 12, 2316–2316 (2011).
32. Knouft, J. H. & Anthony, M. M. Climate and local abundance in freshwater fishes. *R. Soc. Open. Sci*. 22, 160093 (2016).
33. Kulbicki, M., MouTham, G., Vigliola, L., Wantiez, L., Manaldo, E., Labrosrre, P. & Letourneur, Y. *Major coral reef fish species of the South Pacific with basic information on their biology and ecology*. 107. CRISP-IRD report. Noumea SPC.
34. Zimpfer, N. L., Rhodes, W. E., Silverman, E. D., Zimmerman, G. S. & Richkus, K. D. *Trends in duck breeding populations 1955-2012*. United States Fish and Wildlife Service: Publications, Administrative Report (2012).
35. Sauer, J. R., Link, W. A., Fallon, J. E., Pardieck, K. L. & Ziolkowski Jr, D. J. The North American breeding bird survey 1966-2011: summary analysis and species accounts. *North Am. Fauna*. 79, 1–32 (2013).
36. Chytrý, M., Hennekens, S. M., Jiménez-Alfaro, B., Knollová, I., Dengler, J., Jansen, F., Landucci, F., Schaminée, J. H. J., Aćić, S., Agrillo, E. et al. European vegetation archive (EVA): an integrated database of European vegetation plots. *Appl. Veg. Sci*. **19**, 173–180 (2015).
37. Woudenberg, S. W., Conkling, B. L., O'Connell, B. M., LaPoint, E. B., Turner, J. A. & Waddell, K. L. *The forest inventory and analysis database: database description and users manual version 4.0 for phase 2*. Gen. Tech. Rep. RMRSGTR-245. Fort Collins, CO: U.S. Department of Agriculture, Forest Service, Rocky Mountain Research Station (2010).
38. Lüdecke, Danial. ggeffects: tidy data frames of marginal effects from regression models. *J. Open Softw.* **26**, 772 (2018).
39. Tobias, J. A. & Pigot, A. L. Integrating behaviour and ecology into global biodiversity conservation strategies. *Philos. Trans. R. Soc. B. Biol. Sci.* **374**, 20190012 (2019).
40. Sheard, C., Neate-Clegg, M. H. C., Alioravainen, N., Jones, S. E. I., Vincent, C., MacGregor, H. E. A., Bregman, T. P., Claramunt, S. & Tobias, J. A. Ecological drivers of global gradients in avian dispersal inferred from wing morphology. *Nat. Commun.* **11**, 2463 (2020).

41. Weeks, B. C., O'Brien, B. K., Chu, J. J., Claramunt, S., Sheard, C. & Tobias, J. A. Morphological adaptations linked to flight efficiency and aerial lifestyle determine natal dispersal distance in birds. *Funct. Ecol.* **36**, 1681–1689 (2022).
42. Britnell, J. A., Zhu, Y., Kerley, G. I. H. & Shultz, S. Ecological marginalization is widespread and increases extinction risk in mammals. *PNAS* **120**, e2205315120 (2023).
43. Faurby, S., Davis, M., Pedersen, R. Ø., Schowanek, S. D., Antonelli, A. & Svenning, J-C. PHYLACINE 1.2: the phylogenetic atlas of mammal macroecology. *Ecol*. **99**, 2626–2626 (2018).
44. Grames, E. M., Stillman, A. N., Tingley, M. W. & Elphick, C. S. An automated approach to identifying search terms for systematic reviews using keyword co-occurrence networks. *Methods Ecol. Evol.* **10**, 1645–1654 (2019).
