# Supplementary material for: Plants with higher dispersal capabilities follow ‘abundant-centre’ distributions but such patterns remain rare in animals
Source: Nat Commun. 2025 Sep 2;16:8205. doi: 10.1038/s41467-025-63566-0 (PMC12405579; doi:10.1038/s41467-025-63566-0)
Supplement: Supplementary file 2 — Reporting Summary [file 41467_2025_63566_MOESM2_ESM.pdf]

nature portfolio

Corresponding author(s): Connor T. Panter
Last updated by author(s): May 11, 2025

Reporting Summary

Nature Portfolio wishes to improve the reproducibility of the work that we publish. This form provides structure for consistency and transparency in reporting. For further information on Nature Portfolio policies, see our Editorial Policies and the Editorial Policy Checklist.

Statistics

For all statistical analyses, confirm that the following items are present in the figure legend, table legend, main text, or Methods section.

n/a	Confirmed	
☐	☒	The exact sample size (n) for each experimental group/condition, given as a discrete number and unit of measurement
☒	☐	A statement on whether measurements were taken from distinct samples or whether the same sample was measured repeatedly
☐	☒	The statistical test(s) used AND whether they are one- or two-sided<br>*Only common tests should be described solely by name; describe more complex techniques in the Methods section.*
☐	☒	A description of all covariates tested
☐	☒	A description of any assumptions or corrections, such as tests of normality and adjustment for multiple comparisons
☐	☒	A full description of the statistical parameters including central tendency (e.g. means) or other basic estimates (e.g. regression coefficient) AND variation (e.g. standard deviation) or associated estimates of uncertainty (e.g. confidence intervals)
☐	☒	For null hypothesis testing, the test statistic (e.g. F, t, r) with confidence intervals, effect sizes, degrees of freedom and P value noted<br>*Give P values as exact values whenever suitable.*
☒	☐	For Bayesian analysis, information on the choice of priors and Markov chain Monte Carlo settings
☒	☐	For hierarchical and complex designs, identification of the appropriate level for tests and full reporting of outcomes
☒	☐	Estimates of effect sizes (e.g. Cohen's d, Pearson's r), indicating how they were calculated

Our web collection on statistics for biologists contains articles on many of the points above.

Software and code

Policy information about availability of computer code

Data collection	Litsearchr R package (Grames et al. 2019); WebPlotDigitizer version 4.5 (Rohatgi 2021).
Data analysis	All data analyses were performed in R, version 4.0.5 (R Core Team 2021).<br>Packages include:<br>CoordinateCleaner (Zizka et al. 2019)<br>hmisc (Harrell Jr 2022)<br>pheatmap (Kolde 2019)<br>metafor (Viechtbauer 2010)<br>ggeffects (Lüdecke 2018)<br>MuMIn (Barton 2020)<br>rotl (François et al. 2017)<br>phytools (Revell 2024)

For manuscripts utilizing custom algorithms or software that are central to the research but not yet described in published literature, software must be made available to editors and reviewers. We strongly encourage code deposition in a community repository (e.g. GitHub). See the Nature Portfolio guidelines for submitting code & software for further information.

Data

Policy information about availability of data

All manuscripts must include a data availability statement. This statement should provide the following information, where applicable:

- Accession codes, unique identifiers, or web links for publicly available datasets
- A description of any restrictions on data availability
- For clinical datasets or third party data, please ensure that the statement adheres to our policy

Animal and plant data sets are available via the DRYAD Data Repository and can be accessed using the following URL: https://doi.org/10.5061/dryad.zgmsbccj2 (This link is the Reviewer URL and should be updated prior to publication).

Research involving human participants, their data, or biological material

Policy information about studies with human participants or human data. See also policy information about sex, gender (identity/presentation), and sexual orientation and race, ethnicity and racism.

Reporting on sex and gender	NA
Reporting on race, ethnicity, or other socially relevant groupings	NA
Population characteristics	NA
Recruitment	NA
Ethics oversight	NA

Note that full information on the approval of the study protocol must also be provided in the manuscript.

Field-specific reporting

Please select the one below that is the best fit for your research. If you are not sure, read the appropriate sections before making your selection.

☐ Life sciences ☐ Behavioural & social sciences ☒ Ecological, evolutionary & environmental sciences

For a reference copy of the document with all sections, see nature.com/documents/nr-reporting-summary-flat.pdf

Ecological, evolutionary & environmental sciences study design

All studies must disclose on these points even when the disclosure is negative.

Study description	This study represents the most comprehensive test of the 'abundant-centre' hypothesis across geographic space. We explore the effects of dispersal-related species traits and geographic variables on global abundance-distance correlation coefficients for 3,660 species.
Research sample	Research sampling spanned 3,660 species which included 3,060 animal and 600 plant species. Within these taxonomic groups, we computed species-level abundance-distance correlation coefficients for 1,683 birds, 1,131 reef fishes, 202 mammals, 44 freshwater fishes, 386 herbs, 120 trees, 65 grasses and 29 shrubs. Our test of the 'abundant-centre' hypothesis is the largest to date in terms of research sample size and global coverage.
Sampling strategy	Spearman Rank Correlations were used to compute abundance-distance correlation coefficients. These were transformed to Fisher's z-scores for use in all meta-analytical models. For all meta-regression models, we controlled for the influence of species-specific sampling effort by weighting each species by the total number of abundance observations. Grand mean effect sizes were calculated using intercept-only models that included a random term for each effect size. To explore the effects of the species traits and geographic variables on abundance-distance relationships, we expanded the grand mean models and ran a series of mixed-effects meta-regression models by including the trait and geographic variables as explanatory moderators. Separate models were run for animals and plants to account for life history and sampling differences. We explored the effects of combinations of species trait and geographic variables by running a series of interaction models, with the explanatory moderators fitted as interaction terms.
Data collection	A systematic literature search was conducted on 23rd July 2021 by querying the ISI Web of Science database. We optimized our search string using the R package 'Litsearchr' (Grames et al. 2019). In addition to this, we conducted a snowball search of studies that cited the foundational work of Sagarin and Gaines (2002). The lead author designed the data search strategy and extracted the data. Abundance data with associated spatial coordinates were extracted from studies resulting from the TITLE, ABSTRACT, FULL TEXT screening processes. Where data were unavaliable, corresponding authors were contacted via email and were invited to share data. Alternatively, we used the web-based tool WebPlotDigitizer version 4.5 (Rohatgi 2021) to extract data directly from figures. Where possible, abundance and distance data were extracted directly from associated supplementary material from published studies.

	Where distance from range centroid data were unavaliable, we measured the distance from geographic range centres using QGIS version 3.14.16 (QGIS.org 2022).
Timing and spatial scale	Present day; Global.
Data exclusions	Abundance-distance correlation coefficients for nine invertebrate species were excluded from data analyses due to low sample sizes compared to other taxonomic groups. This decision was taken as minimum sample sizes (N = 30) for statistical analyses were not met. Additionally, variables relating to tests of spatial scale on global abundance-distance relationships were also excluded from the statistical analyses. The calculated variable 'extent' was excluded due to uneven and insufficient sample sizes required for robust statistical analyses. The 'focus' variable was excluded due to similarilites in categorisation with the 'grain' variable. Finally, the 'grain' variable was excluded due to being strongly correlated with other explanatory moderator groups: animal taxonomic group and plant functional group.
Reproducibility	This study is not dependent on experimental data, therefore, we did not repeat any laboratory analyses. However, all code and data used in this study will be made publicly available via DRYAD upon publication.
Randomization	NA
Blinding	NA

Did the study involve field work? ☐ Yes ☒ No

Reporting for specific materials, systems and methods

We require information from authors about some types of materials, experimental systems and methods used in many studies. Here, indicate whether each material, system or method listed is relevant to your study. If you are not sure if a list item applies to your research, read the appropriate section before selecting a response.

Materials & experimental systems

n/a	Involved in the study	
☒	☐	Antibodies
☒	☐	Eukaryotic cell lines
☒	☐	Palaeontology and archaeology
☒	☐	Animals and other organisms
☒	☐	Clinical data
☒	☐	Dual use research of concern
☒	☐	Plants

Methods

n/a	Involved in the study	
☒	☐	ChIP-seq
☒	☐	Flow cytometry
☒	☐	MRI-based neuroimaging

Plants

Seed stocks	NA
Novel plant genotypes	NA
Authentication	NA
